# Origin of animal multicellularity: precursors, causes, consequences—the choanoflagellate/sponge transition, neurogenesis and the Cambrian explosion

**DOI:** 10.1098/rstb.2015.0476

**Published:** 2017-02-05

**Authors:** Thomas Cavalier-Smith

**Affiliations:** Department of Zoology, University of Oxford, South Parks Road, Oxford OX1 3PS, UK

**Keywords:** epithelial origin, placozoa, nematocyst, selective advantages of cell differentiation, vendozoa, origin of bilateria

## Abstract

Evolving multicellularity is easy, especially in phototrophs and osmotrophs whose multicells feed like unicells. Evolving animals was much harder and unique; probably only one pathway via benthic ‘zoophytes’ with pelagic ciliated larvae allowed trophic continuity from phagocytic protozoa to gut-endowed animals. Choanoflagellate protozoa produced sponges. Converting sponge flask cells mediating larval settling to synaptically controlled nematocysts arguably made Cnidaria. I replace Haeckel's gastraea theory by a sponge/coelenterate/bilaterian pathway: Placozoa, hydrozoan diploblasty and ctenophores were secondary; stem anthozoan developmental mutations arguably independently generated coelomate bilateria and ctenophores. I emphasize animal origin's conceptual aspects (selective, developmental) related to feeding modes, cell structure, phylogeny of related protozoa, sequence evidence, ecology and palaeontology. Epithelia and connective tissue could evolve only by compensating for dramatically lower feeding efficiency that differentiation into non-choanocytes entails. Consequentially, larger bodies enabled filtering more water for bacterial food and harbouring photosynthetic bacteria, together adding more food than cell differentiation sacrificed. A hypothetical presponge of sessile triploblastic sheets (connective tissue sandwiched between two choanocyte epithelia) evolved oogamy through selection for larger dispersive ciliated larvae to accelerate benthic trophic competence and overgrowing protozoan competitors. Extinct Vendozoa might be elaborations of this organismal grade with choanocyte-bearing epithelia, before poriferan water channels and cnidarian gut/nematocysts/synapses evolved.

This article is part of the themed issue ‘Evo-devo in the genomics era, and the origins of morphological diversity’.

## 1. Introduction: unicells to multicells (and vice versa)

Unicells vastly outnumber multicells and are far more important for the biosphere in biogeochemical recycling. Bacteria and protists greatly exceed vertebrates in different kinds of organism too. Lamarck thought unicells so evolutionarily recent that they had not yet had time to inexorably become multicellular. Not so; they existed billions of years longer than complex multicells and may outlive them. There are hordes of excellent unicellular niches; multicellularity is often selectively disadvantageous. Yeasts evolved multiply from multicellular filamentous ancestors; Myxozoa are parasitic unicells that evolved from animals with nervous systems (early-branching Cnidaria), losing epithelia, connective tissue, nerves and 70% of genes as useless, only their multicellular spores keeping nematocysts [1]. So how and why do some lineages become multicellular? Evolving multicellularity is mechanistically extremely simple. Every unicell group has a cellular and mutational potential to do so given a selective advantage.

Multicellularity evolves in two ways. Naked cells, as in animals and slime moulds, evolve glue to stick together. Walled cells modify wall biogenesis to inhibit the final split that normally makes separate unicells, so daughters remain joined. The ease of blocking that split allowed almost every group of bacteria, fungi and plants (and many chromists) to evolve multicellular walled filaments, more rarely two-dimensional sheets, most rarely three-dimensional tissues. Tissues require more geometric control of daughter wall orientation, as in embryophyte green plants and chromist brown algae; both can grow longer than blue whales. Evolving tissues is selectively harmful to many walled multicells whose filaments are best for reproductive success. Almost all multicells retain unicellular phases (eggs, sperm, zygotes), so adhesion is temporally controlled and developmentally reversible—except for purely clonal vegetatively propagating plants or ‘colonial’ invertebrates (evolutionarily transient) the only organisms that are never unicellular.

Merely joining daughter cells together suffices to create efficient multicellular phototrophs or osmotrophic saprotrophs because their essential trophic features remain intracellular. A bacterial or protist phototroph can easily become multicellular while maintaining the same way of feeding and identical cell functions. An algal filament feeds (on light, H_2_O, CO_2_ and minerals) just as does a single cell; so does a saprotrophic bacterial or fungal filament. However, a phagotrophic amoeba could not aggregate into a multicellular body and still locomote and feed the same way. Nor could most other protozoa. Many amoebae have become multicellular, but only temporarily for spore dispersal, not feeding. Aggregative multicellularity has produced multispore fruiting bodies numerous times in fundamentally different protist lineages (dictyostelids in Amoebozoa, *Guttulinopsis* in Cercozoa, in Ciliophora [2]), so is evolutionarily easy. That is because their multicellular phases are non-trophic; they evolved purely for efficient aerial spore dispersal, free of conflict between need to feed and to aggregate; spores still function as unicells. Thus, *Dictyostelium* [3] is irrelevant for properly understanding animal multicellularity origins.

## 2. Uniqueness of animal multicellularity

If evolving multicellularity is mechanistically so easy for bacteria and protists, then why did animals evolve only once? Primarily, because it is selectively immensely harder for organisms that feed by swallowing others or bits of them (a purely eukaryotic propensity) to switch from intracellular phagocytosis, as in amoebae or ciliates, to eating with a multicellular mouth and gut, whose cells have novel functions and structures absent in their unicellular ancestors. Animal feeding is effective only if novel cell types cooperate at a higher organizational level; most give up the ability to feed or reproduce, huge selective disadvantages not easily overcome.

**Figure 1.**
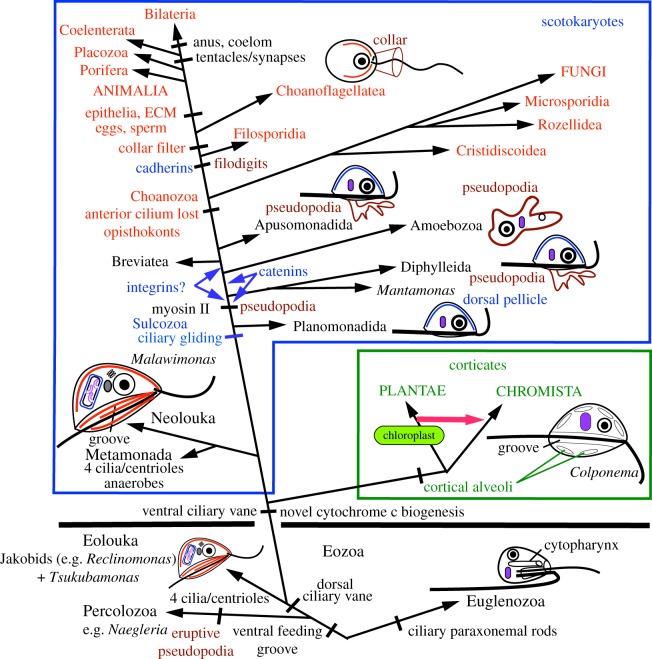
Cell structure divergence in phagotrophic non-amoeboid flagellates provided the basis for evolving animals, fungi, plants and chromists. Pseudopodia evolved secondarily, myosin II providing the basis for pseudopodia in animals, Amoebozoa (and Percolozoa) and muscles. Chloroplasts, originating when the plant ancestor enslaved and modified undigested cyanobacteria, were transferred laterally (red arrow) to make chromists (e.g. brown seaweeds, diatoms, dinoflagellates) whose ancestor modified an enslaved undigested red alga. The most basic eukaryote structural dichotomy contrasts Euglenozoa (parallel centrioles; cilia with paraxonemal rods; cytopharynx for feeding) and excavates (Percolozoa, Eolouka, Neolouka: orthogonal centrioles: no paraxonemal rods; feeding by phagocytosing prey drawn into a ventral groove by posterior ciliary currents). The pre-animal lineage lost excavate groove-feeding by evolving ventral ciliary gliding locomotion to generate Sulcozoa, protozoa with a dorsal proteinaceous pellicle (blue). Irrespective of whether the eukaryote tree is rooted within the protozoan subkingdom Eozoa as shown (most likely) or beside Eolouka-like *Reclinomonas* with the most primitive mitochondria, the immediate ancestors of animals (Choanozoa) arose by loss of the anterior cilium and sulcozoan dorsal pellicle to make opisthokonts (in red) with a radically simplified, more radially symmetric, microtubular cytoskeleton. Long actin-supported filodigits arose in the ancestor of Filosporidia and choanoflagellates and became a circlet of microvilli to make the choanoflagellate/sponge collar for catching bacteria. Filosporidia comprise Filasterea, Ichthyosporea, Corallochytrea [12]. The four derived kingdoms (e.g. ANIMALIA, PLANTAE) are shown in upper case; all taxa in lower case belong to the basal eukaryotic kingdom Protozoa.

In 1866, James-Clark discovered choanoflagellate protozoa and their feeding on bacteria trapped by a collar surrounding their undulating cilium that generates the water current that draws them towards it. He noted that sponge collar cells (choanocytes) have the same structure and feeding method, correctly suggesting that sponges evolved from a choanoflagellate [4]. Often sponges were thought unrelated to other animals, being classified in Protista by Haeckel and Protozoa by Kent [5]. Schulze [6] argued that sponge spermatogenesis allied them with eumetazoa, but doubted the homology of choanocytes and choanoflagellates. For over a century, opinion ebbed and flowed between these contradictory views, until sequence trees proved that sponges are related to other animals and choanoflagellates are the closest protozoan relatives of animals [1,7–9]. Ultrastructurally, collars of both consist of a circlet of microvilli crosslinked by a mucus mesh into an extremely effective bacterial filter; trapped bacteria are moved down to the cell body for phagocytosis [10]. Unaggregated microvilli are present generally on the cell body; choanoflagellate microvilli exemplify a broader class of narrrow cell extensions (filodigits [11]) supported by a tight actin-filament bundle that probably evolved from ancestral opisthokont filopodia in the common ancestor of holozoa, the clade comprising animals, choanoflagellates and Filosporidia (figure 1). Fascin that crosslinks filodigit actin filaments, villin at their base, signalling protein Vav-1 at their tips, myosin X (transporting proteins to their tip), and other functionally related proteins, all originated in the holozoan last common ancestor [13], as did several other myosins [14]; that strongly substantiates the conceptual distinction of filodigits from more generalised filopodia of deeper branching protozoa that lack them as well as filodigits being the key morphological synapomorphy for holozoa [11]. Filodigits, absent from other protists, were presumably present in the immediate ancestors of the first stem choanoflagellates from which animals almost certainly evolved; they apparently evolved at the same time as cadherins that might initially have been involved in holozoan biology long before animals recruited them for epithelial cell adhesion [15]. Of choanoflagellate orders, the mostly surface-attached Craspedida of more primitive morphology and feeding mode are excellent models for stem choanoflagellates also, except for having lost some key animal precursor proteins that remain in more distant protists; planktonic Acanthoecida whose collar filodigits manipulate secreted silica strips into elaborate loricas (enabling a novel filter feeding mode) are highly derived, not directly relevant to animal origins [10].

Among extant animals, only sponges could have evolved directly from protozoa without changing feeding mode. The key problems in understanding animal origins are therefore how and why sponges evolved from a craspedid-like stem choanoflagellate and later generated all other animals. I attempt to explain both after briefly outlining enabling protozoan innovations. I shall emphasize simple conceptual aspects of the choanoflagellate/animal transition, often overlooked but more important than discovering extra protozoan genes suitable as precursors to animal functions. Such ancestral features exist in both choanoflagellates and more distant protozoan relatives of substantially different cell structures and feeding mode [12].

In the light of site-heterogeneous trees using 187 protein sequences [16,17], figure 1 summarizes the major eukaryote clades and key steps in eukaryote cell evolution that paved the way for later innovations that generated animals. Like choanoflagellates, Filosporidia (next most distant animal cousins) belong to the protozoan phylum Choanozoa that ancestrally evolved a swimming mode with a single posterior cilium (i.e. opisthokont—‘posterior oar’ in Greek) like archetypal animal sperm or fungal zoospores that evolved by modifying ancestral opisthokont cell structure [11,16]. Immediate outgroups to opisthokonts are successively more distant branching predominantly biciliate lineages of phylum Sulcozoa that typically move not by swimming but by gliding on surfaces by ciliary surface motility propelling one semi-rigid cilium and feed by emitting newly evolved, bacteria-grabbing, branching pseudopodia from the cell's ventral ciliary groove [11,16].

Sulcozoan flagellates clearly could not have retained their characteristic locomotory or feeding modes had they evolved glue to stick together as a multicellular organism; such mutants would necessarily quickly starve to death. Nor could their immediate ancestors—three successive groups of swimming, not gliding flagellates (i.e. Neolouka, Eolouka, Percolozoa) collectively called excavates because their ventral groove looks more obviously scooped out [11,17]. The groove phagocytoses prey propelled therein by both cilia, the posterior often having one or two lateral vanes to increase its thrust. Their ancient groove-supporting asymmetric cytoskeleton, with five distinct microtubular ciliary roots and many characteristic filaments, was inherited by Sulcozoa, initially with diverse minor modifications, but radically simplified and made more symmetric during the origin of the opisthokont body plan by anterior ciliary loss, possibly in association with a protochoanoflagellate feeding mode [11].

Knowing the structure and evolutionary potential of the closest relatives and ancestors of animals (figure 1) and that opisthokont cells were radically simplified compared with their ancestors does not directly explain animal origins, but helps distinguish central from peripheral aspects of the process and avoid pitfalls from erroneous assumptions about ancestors. Most things we inherit from our unicellular ancestors evolved before the excavate/Euglenozoa split. Only a few arose within the scotokaryote clade that embraces opisthokonts, Amoebozoa, Sulcozoa and Neolouka, and is sister to the cytologically substantially different plant/chromist clade (Corticata) [17].

Integrins and associated molecules used for epithelial cell adhesion to extracellular matrix (ECM) were secondarily lost by choanoflagellates and fungi; without full genomes for the deepest branching Sulcozoa, the exact point of origin is unclear (figure 1): though not yet known for branches before Breviatea, integrins might have arisen earlier with scotokaryote pseudopodia, for mediating reversible adhesion to the substratum and/or pseudopodial actin bundle attachment/assembly via talin/vinculin that certainly evolved earlier [12], at least prior to Amoebozoa. If, instead, integrins help actin attachment to sulcozoan dorsal pellicles, they possibly arose one node earlier. Determining intracellular distribution and functions in early Sulcozoa would clarify the integrin adhesion system's original functions; as genomes are known only from very simplified and derived Amoeboza lacking integrins, they are also needed for early diverging Amoebozoa with more complex extracellular coats/thecae [18] that might involve integrins. Though lacking typical integrins, *Dictyostelium* has a β-integrin-like adhesion protein [19] and its multicellular prefruiting 'slug' evolved ahaerens-like junctions involving preexisting actin-filament-binding catenins [20] (convergently with independently evolved animal adhaerens junctions) but unlike sponges and other animals could not recruit cadherins as they only evolved later (with filidigits in ancestral holozoa) [12].

On present evidence, excavates and Sulcozoa, successive ancestors of Choanozoa, never evolved multicellularity, nor did any Choanozoa except choanoflagellates whose unique cell structure and feeding mode preadapt them for evolving multicellularity. Therefore, discovery in non-choanoflagellate Choanozoa and Sulcozoa of integrins and of cadherins, and synaptic proteins and other neural channel proteins in choanoflagellates and filosporidia [15,21], does not explain how animals originated. It tells us (unsurprisingly) that pre-existing proteins were recruited for the job and diversified by gene duplication and divergence (standard for any substantial innovation) but not why these protozoa failed to become animals and only one lineage did. We must identify selective forces that make it impossible for most protists to evolve a body with a gut and explain why only one lineage ever did. I contend that it was not the presence of potential glue molecules, but the rare ability of choanoflagellate cells to stick together yet still feed as before that made stem choanoflagellates our ancestors. Inability to do this would strongly select against similar aggregative mutations in other groups.

## 3. Choanoflagellate and flagellate multicellularity

In choanoflagellate colonies, every cell can feed. To become a sponge, the majority must abandon feeding as collar cells, lowering feeding potential dramatically. A sponge could evolve only if a body were made where reduction in feeding capacity caused by a lower ratio of feeding to non-feeding cells was more than compensated by an indirect increase in feeding or survival efficiency. For understanding animal origins, the key problem is not how cells evolved a capacity to stick together (trivial)—or even why—but defining the selective forces that promoted the fundamental differentiation between sponge feeding cells (choanocytes) and non-feeding cells and between cells that stick together as epithelia and connective tissue cells embedded separately in a gelatinous mesohyl. Did epithelia evolve first or did epithelia and mesenchyme coevolve?

Four different ways of making multicellular choanoflagellates exist. Many become ‘colonial’ sessile organisms by evolving thin extracellular stalks that join cells together to form branched tree-like structures analogous to corals or plants [5,10]. Other flagellate groups also evolved multicellular sessile lineages with branching stalks; many heterotrophic, e.g. biciliate bicoecids (heterokont chromists), pseudodendromonads (heterokont chromists), sessile ciliates (e.g. *Carchesium*, *Zoothamnion*); some algal, e.g. chrysophyte *Dinobryon*. Mucilaginous multicellular branching structures are formed by *Rhipidodendron* (cercozoan chromists) or *Phalansterium* (uniciliate Amoebozoa). As no branching protists evolved a multicellular tissue, similar ‘colonial’ choanoflagellates are probably not directly relevant to animal origins. Nonetheless, they show that various linked flagellates can still feed in the same way as when unicellular, and their frequency suggests that branching stalks advantageously enable them to sweep prey from a much larger water volume than can one sessile cell. Filtering more water by a different sessile body form is, I argue, the selective advantage that made sponges.

More rarely, choanoflagellate multicells arise by linking adjacent cells by their collar microvilli as in *Proterospongia choanojuncta*, but I doubt this had a potential to yield a sponge. Sponge collars also join laterally often by a second mucus mesh to achieve 100% removal of suspended bacteria [22], showing intercellular cooperation efficacy.

The loricate *Diaphanoeca sphaerica,* where cells often clump in hollow balls with cilia pointing inwards [23], exemplifies a third multicell type incapable of progressing to a tissue. Comparing this with a sponge choanocyte chamber [24] was misleadingly superficial as *Diaphanoeca*, like other loricates (Acanthoecida), are tiny cells suspended within a much larger lorica of siliceous strips porous to water currents carrying prey. Aggregating porous loricas by connecting longitudinal strips allows colonial feeding despite cilia pointing inwards, as the collar outer surface that traps food still faces outwards. Water and bacteria can pass through the lorica mesh or wide interlorica spaces, so feeding mode is unchanged compared with unicells; cell bodies are not in contact so could not evolve into an epithelium to make a sponge. Acanthoecida are necessarily an evolutionary dead end.

Non-loricates (Craspedida) never aggregate with cilium facing inwards like sponges as that would suicidally stop collar-based feeding. *Sphaeroeca* is a multicellular planktonic craspedid whose colonies are hollow balls with a surface cell monolayer, associated by cell bodies not collars, analogous to the alga *Volvox* that Hardy [25] invoked as a potential animal ancestor because of its simple feeding. The craspedid *Salpingoeca rosetta* reversibly makes little multicellular balls, a capacity influenced by bacteria [26]. Numerous other flagellates, e.g. chrysophyte chromists, evolved similar free-swimming multicell balls. These would be incapable of progressing towards a multilayered Haeckelian gastraea, because gastrulation-like internalizing cells would prevent their feeding, without immediate benefit, and thus be strongly disadvantageous. However, by settling on stable surfaces as sessile filterers, they would encounter new selective forces favouring cell differentiation, enabling animal origin. Sponges evolved thus from a craspedid-like stem choanoflagellate.

## 4. Evolving a triploblastic presponge

**Figure 2.**
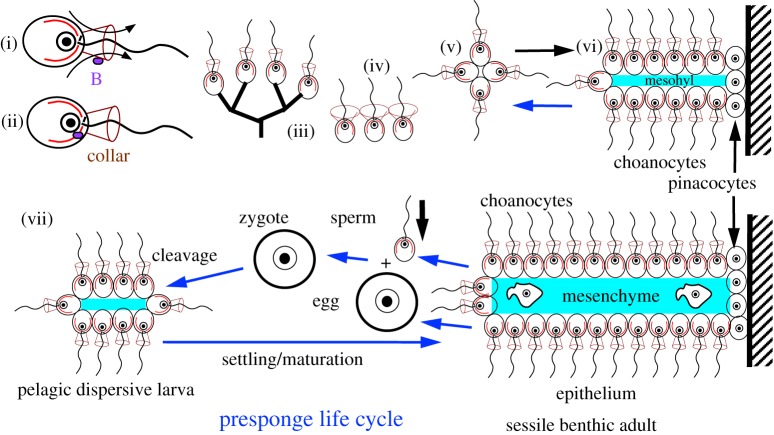
Evolution of an archetypal animal, a presponge (vii), from a stem choanoflagellate (i–ii,v) prior to integrin loss by crown choanoflagellates. Choanoflagellates feed by catching bacteria (B) drawn by ciliary water currents (i, arrows) to their collar filters; the cell body phagocytoses them (ii). Extant craspedid choanoflagellates may be unicells (i,ii) or daughter cells may stick together by branched stalks (iii) or collar microvilli (iv) to make sessile multicells or via cell bodies to make planktonic swimming balls of cells (v). The first animal could simply have evolved (horizontal black arrow) by such a ball of cells joined laterally by cadherins settling onto a rockface (cross-hatched), differentiating non-ciliated pinacocytes for attachment and for support secreting extracellular mesohyl (turquoise) by both cell types and attached to them via pre-existing integrins (vi). This simplest presponge presumbly budded off ciliated swimming balls for dispersal (blue arrow), and probably had to evolve nutrient transfer from choanocytes to pinacocytes. (vii) Competition for filtering larger water volumes led to larger, stronger, three-layer (prototriploblastic) feeding laminas with mesenchyme cells specializing in ECM secretion sandwiched between choanocyte epithelia. Larger laminas led to divergent selection for large eggs capable of rapid cleavage and more numerous smaller sperm, both originally differentiated from choanocytes (rightmost blue arrows). As size increased, the pluripotent nonciliated mesenchyme cells differentiated into proliferative stem cells (archaeocytes: thenceforth the usual precursors of eggs, choanocytes continuing to generate sperm) and terminally differentiated cells (lophocytes) secreting collagen fibres to increase mechanical strength.

Willmer emphasized the basic dichotomy between ciliated epithelial and non-ciliated, amoeboid, connective tissue cells as fundamental to animal development [27]. Figure 2 summarizes a potential pathway by which a stem choanoflagellate lineage, initially a standard swimming ball of choanocytes, could transform into a sessile precursor of sponges by evolving comparable somatic cell differentiation to anchor itself to a rock. The new cell type was a basal non-ciliate anchoring cell that secreted ECM—effectively a basal pinacocyte. An ECM of mucopolysaccharide and collagen would form a supportive mesohyl skeleton between two monolayer sheets of choanocytes—the ancestral choanoderm. The selective advantage of this novel three-layer structure would be filtering food from a much larger volume of water, just as branching colonial choanoflagellates do. ECM support would allow a much larger structure that could overtop simple branched choanoflagellates with choanocytes only. This could have increased food caught by choanocytes more than enough to compensate a presponge for loss of filter-feeding capacity by basal pinacocytes and ECM secretion costs. If so, selection for taller, wider multicellular filters processing larger volumes of seawater would immediately unavoidably ensue. Flow hydrodynamics for maximizing catch and architectural principles maximizing support and filter area would impose novel selective forces yielding similar structures to bivalve mollusc gills. Pinacocytes would develop contractile actomyosin and surface adhesion analogously to an amoeba to spread flattened extensions and cell contacts over the holdfast portion of the sessile lamina with least cost. They retained a capacity for phagocytosis, thus providing a primitive immune system by digesting potentially invasive bacteria for which mesohyl was a nice habitat and food.

The primary dichotomy between uniciliate choanocyte and non-ciliate pinacocyte is also mirrored by that between sperm and egg. Therefore, part of the same gene switches needed for somatic differentiation could also be used to differentiate gametes. Once a three-layered structure with just two somatic cell types evolved, presponges could become quite large (compared with choanoflagellate unicells); selection for rapid establishment of a large embryo would strongly favour oogamy (large egg and numerous small sperm) by modifying choanocytes, presumably hermaphrodite. The animal bauplan was in place once a selective force for ever-larger filtering structures built from two dissimilar cell types existed: two germ line and two soma cell types. Accidental fragments could also reproduce vegetatively as choanocytes retained pluripotency [28]. There was no necessary sacrifice of reproductive potential as in *Dictyostelium* dead stalk cells.

Another selective advantage of evolving mesenchyme and massive tissues perhaps gave extra impetus to early animal evolution. Mucilage easily harbours bacterial symbionts potentially able to provide enough extra food to repay a presponge several times over the trophic and reproductive costs of non-feeding cells. Cultivating cyanobacteria in ECM mucilage would make the photophagotrophic consortium an extremely effective competitor with merely branched choanocyte-only colonial choanoflagellates. Lichen fungi can survive solely by cultivating cyanobacteria; a presponge could be even better off, being also a phagotroph able to grow far faster than a lichen in bacteria-rich water. Great Barrier Reef sponges 1–2 m high are often red through being packed with cyanobacteria whose biomass is greater than that of the sponge cells. Lake Baikal giant freshwater sponge tissues cultivate green algae. Both habitats are oligotrophic, making internal algae especially advantageous, but even in habitats rich in particulate food, the majority of sponge species are often photosynthetic [29]. In organic-rich habitats, a presponge could probably eat enough bacteria to subsist without growing algae. Even sponges without cyanobacteria or green algae have a huge bacterial symbiont biomass, often in special bacteriocytes, presumably providing trophic or other advantages such as antibiotic defence against invaders [30]. All choanoflagellates live with bacteria of many kinds. Choanoflagellate–bacteria interactions other than simple predator–prey must affect modern choanoflagellates [31], but could also have had a role in animal origins [32]. Making a tissue without cell walls invites others to eat it; before bilateria, enemies were mainly microbial.

Extra cell types could be added relatively simply to help presponges to grow bigger and be less susceptible to environmental damage. An individual could grow basally across a rock and erect multiple laminae. Spatial controls evolved to prevent laminae from interfering with each other. Presumably, various morphologies and arrangements and ratios of the two basic cell types were experimented with, giving different compromises between maximizing feeding and mechanical stability. An early innovation necessary for large structures was to increase the ECM-synthesizing cells initially perhaps by evolving a third cell type—the ancestral archaeocyte that left the epithelium, entering the mesohyl for secreting ECM in all directions, making a triploblastic tissue with mesenchyme sandwiched between two epithelia. Nowadays archaeocytes and choanocytes are the demosponge stem cells, expressing PIWI double-strand RNA-binding domain proteins whose short-RNA related functions are associated with germline and stem cell maintenance in higher animals [28] as well as with RNAi and chromatin dynamics [34]. Generally, sperm arise from tiny choanocytes and eggs from many-fold larger archaeocytes [35]; possibly therefore the non-ciliated archaeocytes originated from ancestral choanocytes as egg precursors independently of non-ciliated terminally differentiated pinacocytes that like spicule-forming sclerocytes and other non-stem cells do not express PIWI proteins. Very likely PIWI suppression in pinacocytes arose in the ancestral presponge with only two somatic cell types when its ancestral function of protecting proliferating cells from transposons (that goes back even to prokaryotes) became unimportant in the very first dead-end somatic cells.

## 5. Defects of some other scenarios

Site-heterogeneous multigene trees (technically the best) maximally support choanoflagellates being sisters to animals [1,33]; they never branch within or as sister to sponges, as the implausible idea that choanoflagellates evolved reductively from sponges [36] requires. Myxozoan parasites having become somatically unicellular (spores are multicells with uniquely cnidarian nematocyst minicollagens [37]) is one of many examples of selectively comprehensible gross parasitic reduction, but does not make such selectively incredible drastic simplification of a free-living sponge even remotely likely and should not have been cited in its favour [36].

Site-heterogeneous multigene trees equally strongly show sponges as a clade, disproving Nielsen's assumption that eumetazoa are more closely related to homoscleromorphs than others, and invalidating his twin assumptions that ancestral animals were lecithotrophic and eumetazoa secondarily lost lecithotrophy [24]. His suggestion that the first event in animal evolution from a spherical choanoflagellate colony was evolving internal non-ciliate, non-feeding cells to make an ‘advanced choanoblastea’ exemplifies selectively untenable Haeckelian idealistic morphology; such a change would drastically sacrifice feeding potential with no positive benefit and be quickly eliminated by competition. It has been insufficiently recognized that evolving a non-germline soma is not inherently advantageous, but a severe reproductive cost that has to be offset by an extremely strong novel selective advantage. Had a ‘choanoblastaea’ been advantageous, such two-layered pelagic choanoflagellates should still exist; their supposed sponge descendants occupy a separate adaptive zone, so would not have competitively eliminated them as happened for the selectively plausible sessile intermediates of figure 2. The key innovatory sessile benthic stage (vi) of figure 2 provides a definite selective advantage for presponge non-feeding cells, unlike a pelagic choanoblastaea.

## 6. Evolving a water-pumping sponge

This presponge was not a sponge, for it lacked an aquiferous system (AS) with incurrent pores (ostia) and larger excurrent osculum or oscula. AS architecture has two advantages: (i) it increases food supply by pumping much larger water volumes past the choanoderm; (ii) compared with the essentially ‘free-living gills’ of the presponge, placing the choanoderm inside a globular or encrusting body protects choanocytes from damage by sand and other things swept against them by vigorous water currents and from damage by the currents themselves. Essential innovations making a sponge were (i) controlled formation of ostia of appropriate size, frequency and distribution; (ii) rearrangement of pinacocytes and choanocytes to internalize the latter, make a more compact less easily damaged body, and optimize water flow through internal choanoderm-lined channels. Ostia are intercellular in all Homoscleromorpha and most demosponges, but are formed by channels through specialized porocytes in Calcarea and not obviously homologous contractile porocytes in a few haplosclerid demosponges. I suspect they originated not by evolving a new cell type but by spatially controlling pinacocyte contacts and geometry; porocytes evolved later independently in Calcarea and haplosclerids. If so, ostia arose as part of the supracellular rearrangements that made an axially polarized water channel system. This major innovation almost certainly depended on prior evolution of morphogen gradients and homeobox and other spatially controlled switch genes that sponges share with Eumetazoa [22,38]. Benefits of an effective AS might have been the prime driving force for the evolution of animal ‘head/tail’ polarity—nothing to do with heads or tails: the Wnt anterior–posterior axis system probably controls sponge AS development [39,40]. More likely, Wnt axial gradients arose earlier still in vendozoan presponges.

**Figure 3.**
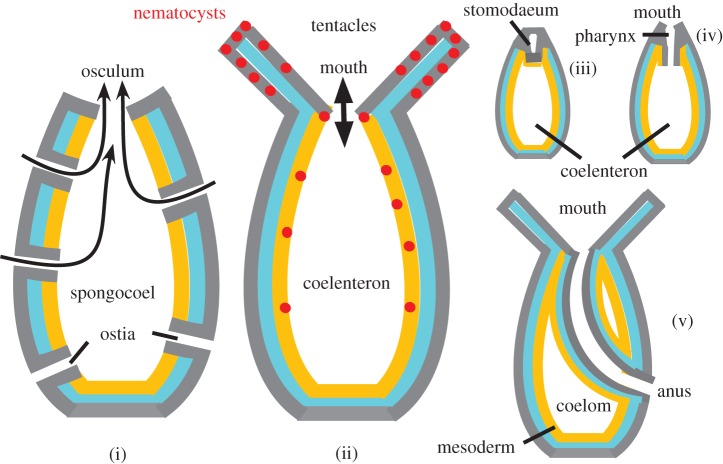
Origins of sponges, Cnidaria and bilateria with homologous body axis polarity. (i) Internalizing presponge choanoderm (yellow) by overgrowth of the pinacoderm (grey) and epithelial rearrangement into an asconoid body form, with incurrent ostia and exhalent osculum, could have established the sponge body plan without adding new cell types. The key innovation may have been Wnt axial prepatterning translated into spatially controlled differentiation by homeodomain transcription factors. (ii) Origin of perioscular and septal nematocysts for catching larger food and tentacular growth led to loss of ostia (as convergently in carnivorous sponges), so spongocoel became coelenteron and osculum the mouth; pre-existing neurotransmitter-secreting cells made synapses (elaborations of cadherin-joined foci) with nematocytes, sensory cells, tentacular and perioral myocytes and each other, making a nerve net controlling feeding behaviour of this stem anthozoan. Not shown is that nematocytes probably originated aborally from sponge flask cells slightly earlier to improve larval settlement and ancestral cnidaria probably evolved pharnyx with bilaterally symmetric ciliary feeding currents and nematocyst-rich octomerous septa for trapping food for extracellular digestion before tentacles (see §8). In some anthozoan polyps, pharynx and coelenteron develop as separate cavities in solid tissue masses (iii); mouth and pharynx/coelenteron connection form by secondary channels opening later (iv). Bending the elongated pharyngeal primordium laterally to fuse basally with the body wall before the lower channel opened could make anus and coelom in one step (v); former endoderm becomes the coelomic and stomodaeal epithelium the gut lining.

Making AS development and functioning more efficient probably entailed differentiating pinacocyte subtypes: specialization of some as myocytes to exert some control on oscular and ostial opening; and multiplication of non-epithelial mesohyl cell types. The branched mesohyl cells that synthesize a variety of neurotransmitters are obvious candidates for precursors of eumetazoan nerve cells, requiring only the origin of electrosensitve channels to cause action potentials and synapses to make a nerve net. The syncytial body form and calcium/potassium action potentials of hexactinellid glass sponges are secondary, not the ancestral condition for sponges, as hexactinellids are related to demosponges not the deepest lineage [1]. They are therefore not directly relevant to origins of animals, sponges or eumetazoa. It was long overlooked that sponges of all four classes are contractile, as contractions are typically slow, taking 15 min to hours; in demosponges pinacoderm mediates this [41]. Many are in constant motion, contracting ostia and water channels and relaxing body parts to modulate pumping [42]. Sponge behaviour primarily involves water-filtering and protection against damage by larger particles or storm surges, but can be adapted to seasonal temperature changes, increased suspended sediment, or even spawning by other sponges or used to expel wastes by ‘sneezing’. It is untrue that they lack sense organs [22]. All have non-motile oscular sensory cilia that use calcium channels for behavioural control [43]. Calcium control of ciliary reversal is well studied in *Chlamydomonas* and could be a general property of eukaryote cilia that evolved during the origin of two structurally and behaviourally dissimilar cilia in the eukaryote cenancestor [44]. In the demosponge *Ephydatia*, oscular sensory cilia lack the centre-pair microtubules as in eumetazoan sensory cilia [43]. Early sponges likely had a solenoid body form [38] with a higher ratio of choanocytes to non-feeding cells than the simplified asconoid of figure 3.

The greater complexity of true sponges over presponges required planktonic ciliated larvae for dispersal to new fixed sites that grew big enough to transform immediately into a tiny triploblastic sponge with internal choanoderm able to feed at once. Abundant egg yolk enabled more rapid development than feeding by surface choanocytes, making sponge larvae lecithotrophic unlike planktotrophic presponge and ancestral eumetazoan ciliated larvae. Larvae evolved phototaxis using cryptochromes [45], not rhodopsin as in eumetazoa; some respond to gravity and have behaviour of similar complexity to eumetazoan larvae with nerves. Like rhodopsin, calcium control of cell behaviour first evolved in eubacteria [46,47] not stem eukaryotes [48], which simply adapted it for the control of actomyosin that evolved in association with bacterial wall loss and the origin of phagotrophy and endomembrane system [44,49].

The phrase ‘from amoeba to man’ epitomizing Haeckel's early phylogenetic views doubly misleads. Amoebae are not primitive but arose from zooflagellate ancestors independently in each of the three ancestrally biciliate eukaryotic supergroups [18]. Epithelial polarized vesicle secretion selectively to apical and basolateral membranes, fundamental to animal organization, is prefigured in zooflagellate cell polarity that may hold molecular clues to its origin [50]. Subcellular differentiation merits intensive study in choanoflagellate models, including spatial differentiation of ciliary and cell membrane proteins. Very likely, membrane protein targeting also differs between intra- and extracollar regions and for microvilli. Selective protein targeting to different membrane regions must have evolved with cilia, exemplified, in exquisite detail, by cytoskeletal architecture and membrane protein targeting to the ciliary pocket of trypanosomes [51], which are Euglenozoa as far from us on the tree as can be (figure 1), showing eukaryotic cell asymmetry's antiquity. Understanding asymmetric cytoskeletons and spatial control of membrane protein secretion of the whole spectrum of zooflagellates as well as sponges will do far more than genomics for elucidating the physical forces that made animals. Making animals is a four-dimensional, not a one-dimensional problem. Many cytoskeletal protein sequences evolve rather fast and have numerous confusing paralogues, not lending themselves easily to one-dimensional bioinformatics. We need phylogenetically informed molecular cell biology with a developmental slant of the form-generating molecules (and their three-dimensional structure, a huge crystallographic challenge) to understand cell morphogenesis, the basis for animal bodies and nervous systems. To learn about learning, we must understand molecular bases of neuronal form, prefigured in branching sponge cells and synapse dynamics. Unlike stem choanoflagellates, merely temporarily polarized aciliate amoebae never evolved into Hacekel.

## 7. Zoophyte^1^ origin of eumetazoa and the nervous system

The larger larvae of true sponges provided a novel, hitherto unexploited, food for predators. One stem sponge lineage, I suggest, evolved nematocysts to catch and digest them, thereby becoming the ancestor of coelenterates (Cnidaria, Ctenophora), a clade on the best multigene trees [33]. Nematocyst discharge of ECM [54] anchors the aboral pole of settling cnidarian planula larvae [55] just as do secretory flask cells at the aboscular pole (similarly anterior when swimming) of sponge larvae [56]. Flask cells are the only larval sponge cell type to coexpress the majority of post-synaptic protein homologues [57], so I suggest, evolved directly into nematocytes by evolving capsular/tube minicollagens [58] and cnidoin elastomer that facilitates their nanosecond discharge [59]. Nematocytes are not independent effectors [60] but innervated by chemical synapses (responsive to glutamate and GABA (γ-aminobutryic acid) in *Hydra* [61]), and thus post-synaptic effectors. I suggest their primary function was to mediate larval settlement and their more complex feeding role evolved only after synapses first evolved between sensory cells and nematocysts and were then secondarily formed with muscles and probably simultaneously with larval ciliated cells, improving adult feeding and larval guidance. If so, chemical synapses arose to facilitate rapid concerted ECM discharge by the aboral cluster of secretory cells that cnidarian and sponge larvae share. Sponges already had glutamate, GABA and NO control of behaviour [62], and synaptic proteins had polarized secretory functions as early as the ancestral unicellular holozoan [21]; very few synaptic proteins are restricted to animals with synapses, choanoflagellates have many [63]. As *Trichoplax* (unlike sponges) has numerous presynaptic protein precursors as well as gap junctions, chemical and electrical synapses probably both originated after the pre-cnidarian lineage diverged from placozoa yielding an anthozoan-like stem coelenterate. Thus, neither muscular [60] nor ciliary control [64] initiated neurogenesis, but neurosecretion, the third, underappreciated universal effector.^1^

Key to neurogenesis was a multicellular precursor with neurotransmitter-making cells and already adjacent receptor and effector cells linkable by evolving synapses under a strong selective advantage, exactly as this flask cell to nematocyte transition postulates without missing links or improbable events. Thus, improving the sessile zoophyte lifestyle by increasing survival (e.g. against waves tearing settling larvae from rocks) at the crucial, but uniquely vulnerable, pelagic–larval/benthic–adult transition was, I contend, the selective force for evolving synapses, ultimately leading to brains, culture and science. Synapses evolved to make ciliated larval settlement faster and more effective by neural coordination of concerted banks of nematocysts under the control of ciliated sensors that selected the best sites. Flask cell precursors concentrate at the aboral pole. Nematocysts remain there to mediate settlement but concentrated also around the osculum (making it a mouth) and along ancestral anthozoan protosepta to trap food.

Adding synaptic junctions not only between sensory cells and nematocysts, but between sensory cells and branched pre-existing branched transmitter-making cells (making internuncial neurons) and myocytes, would establish local neuromuscular control by a nerve net. This speeded oscular contraction making it an effective mouth, its reversible closure plus adhaerens junctions being key innovations for initial extracellular digestion of larger prey caught by oral and septal nematocysts. Having established neuromuscular synapses, pre-existing voltage-dependent Na^+^ and K^+^ channels (both originating in bacteria) were modified to generate sodium/potassium action potentials in longer nerve cell branches for distant coordination of feeding responses, making eating more efficient—a selective advantage an automatic corollary of this explanation of synaptic origin. Action potentials evolved many times, thus easily—not only in hexactinellids, but also filamentous fungi, plants and ciliate protozoa [65]. Axons easily evolved by centrosomally directed cytoskeletal elongation. During gradual changeover, choanocytes and nematocysts could both be used for feeding: no traumatic hopeful-monster, but simple gut evolution from ideal precursors. Catching larger prey was made more efficient by circum-oscular projections evolving into stem anthozoan tentacles. Pre-existing myocytes contracted tentacles to place the prey inside the osculum for better absorption.

Before tentacles evolved, partially redirected ciliary currents (importing food and exporting waste through the mouth) likely made an asymmetric single-siphonoglyph protopharynx; and eight functionally complementary nematocyst-rich septa (arguably modified from internal projections within a sponge of more complex AS morphology than the figure 3 asconoid depicted for simplicity) trapping food within the incipient gut evolved octomerous bilateral symmetry in the ancestral coelenterate. Much later a few demosponges convergently evolved carnivory, some like Cnidaria losing choanocytes and AS [66], without nematocysts or nerves, showing they can evolve carnivory, but carnivory *per se* does not make nerves.

Without giving reasons, Nielsen unjustifiably asserted ‘it seems impossible to derive eumetazoans from an adult sponge’ [24, p. 148]. On the contrary, to evolve a coelenterate from a stem sponge depended on preexisting epithelial adhaerens junctions, enabling extracellular digestion [24], thus converting spongocoel into gut lumen; and required only two key cellular innovations: secretory nematocysts for enhancing larval settling and trapping metazoan prey; action potentials in protoneurons; as well as loss of choanocyte collars and ostia. Neither is mechanistically or selectively unlikely; given copious sponge molecular precursors and complex homologous axial triploblastic organization, both key innovations would have been evolutionarily far easier than origins of either presponges or sponges, so coelenterates should have evolved essentially immediately after sponges, which fits the fossil record. A flask-cell/nematocyst transition makes a cnidarian more simply than Nielsen's assumption of neotenous conversion of a lecithotrophic homoscleromorph sponge larva into a planktotrophic eumetazoan larvae that added an entirely novel adult sessile stage by loss of the whole sponge adult, which did not explain how or why nematocysts originated or how they were linked with synaptic origins. His scenario is far more complex and less plausible selectively than nematocysts converting stem sponge larvae to planulas and switching adults from bacterial to metazoan prey. Nielsen [67] correctly argued that ciliated larvae were present in ancestral eumetazoa and later independently lost by those lacking them, but basal sessile eumetazoan adults did not, as he supposed, evolve from them independently of adult sponges. Thus, the ancestral animal life cycle was a non-Haeckelian alternation of feeding planktonic larvae and sessile feeding adults, lecithotrophy and direct development being multiply derived. Neoteny (accelerated sexual development) probably did occur in the independent of origins of *Trichoplax* (gut loss by secondary flattening when switching to benthic feeding after adhaerens junctions and gap junctions, but before tentacles/neurons, evolved) and Ctenophora.

## 8. Coelenterate unity and diversification

I have argued that the ancestral coelenterate was a bilateral octomerous stem anthozoan that lost choanocyte microvilli as neurally controlled nematocyst/tentacle feeding on larger prey improved, its mouth evolving from the osculum, and ostia closed (except for a pore at the body base in many anthozoa; several in ctenophores) suppressing water channels, yielding a single body cavity, the coelenteron. Choanoderm and endoderm are homologous [68], as are larval swimming with anterior sensory cilia and posterior aboral settlement, and Wnt signalling patterns specifying oral–aboral axes and nervous systems in eumetazoa, including coelenterates [69–71] and sponges [40].

Contrary to dogma, Anthozoa, Scyphozoa and Cubozoa are mostly triploblastic with true mesoderm [72–74]. The Huxley/Haeckel idea that diploblasty preceded triploblasty is wrong. Huxley invented the diploblast concept for Hydrozoa, the only true diploblasts [73]. Phylogenetically they nest deeply within Cnidaria as sister to the triploblastic jellyfish, together making clade Medusozoa [1]. Medusozoa arguably originated by an early anthozoan evolving vegetative scyphistoma-like transverse budding to make planktonic tentaculate forms that could disperse and feed immediately as a medusa without needing metamorphosis from a planula. Sponge and anthozoan larvae and large planktonic protists were probably its initial prey, but as bilateria evolved giving larger necton Scyphozoa and Cubozoa diversified nematocysts and poisons for larger more active prey, but Anthozoa typically kept to smaller snacks, developing large individual polyps (sea-anemones) or most often spreading multipolyp modular body forms and reef formation with dinoflagellate photosynthetic symbionts in oligotrophic waters. Hydrozoa focused on a branching hydroid form with only tiny dispersive medusae and simplified both by narrowing the mesogloea, so became diploblastic. Hydromedusae swim by jet propulsion via whole body contraction that may have been mechanically favoured by extreme mesogloeal thinning through losing mesenchyme cells.

Probably before Medusozoa originated, a stem coelenterate switched completely from benthic to planktonic life by evolving multiaxonemal macrocilia and comb plates (with reversible beat, but swimming typically with mouth anterior, opposite to medusae) and losing nematocysts no longer required for settlement and accelerating oral and sexual development. This radical shift in adaptive zone and developmental fate of the ancestral planula larva entailed numerous unique innovations giving Ctenophora such a different body form from crown cnidarian adults, and unique embryology. In Cnidaria, the larval nervous system is concentrated largely aborally but degenerates during metamorphosis after settlement, being replaced by an oppositely polarized adult system with an oral focus [75]. Unsurprisingly, by eliminating settlement and metamorphosis, ctenophores retained the originally larval neural organization, uniquely developing the statocyst as a neural focus. Ctenophore homologies should be sought with transient larval cnidarian, not adult nervous systems. Although most larval cnidaria lack mouths, some anthozoan larvae have them—accelerated developments independent of the profound ctenophore neoteny.

Ideas that the nervous system evolved twice or was lost by sponges [54–56] are unwarranted [76]. The long ctenophore stem on sequence trees suggests episodic evolutionary hyperacceleration that probably largely erased true phylogenetic signal, allowing slight systematic biases summed over many genes to place them (arguably misleadingly) often below sponges [1], not as sister to Cnidaria as some good trees [33] and organismal characters favour. Complex character loss is far easier than gain; Myxozoa, somatically secondarily unicellular parasites once wrongly considered Protozoa, lost their nervous system, being robustly phylogenetic sisters to *Polypodium*, a tentaculate triploblastic polypoid cnidarian (class Polypodiozoa) whose highly modified planula endoparasites sturgeon oocytes [1]. *Polypodium* triploblasty supports treatment as a separate class outside diploblastic Hydrozoa [77], their actinula-like stolonoid parasitic phase suggesting that the Myxozoa/*Polypodium* clade might be sister to Hydrozoa, as some trees indicate [77]. Myxozoan branches on multiprotein trees that show them as sister instead to all other Medusozoa [1] might have put them artefactually one node too deep, but they could reasonably be genuine sisters of all Medusozoa, as the polyp-like free-living adult has an apparently primitive nerve net [77], as expected if its ancestor evolved directly from a stem anthozoan and its traditional assignment to Medusozoa were incorrect.

Others advocate one neural origin and invoke tree artefacts, giving more supporting details [78]. Saying synaptic origin ‘might occur more than once during ∼600 million years of animal evolution’ [79, p. 607] is 100-fold misleading; fossil ctenophores and cnidaria originated essentially simultaneously (Ctenophora 540 Ma [80]; Cnidaria 560 Ma [81]) as sequence trees' close branching and poor resolution confirm. It was no coincidence; benthic nematocystous anthozoa and pelagic ctenophores likely diverged within 5 Ma of synaptic origin (a complex arguably unique innovation), divergently perfecting benthic tentacular feeding (Cnidaria) or pelagic ciliary current feeding (Ctenophora) by amplifying and recruiting partially different subsets of the choanozoan/sponge protein repertoire. Cambrian ctenophores lack tentacles and have more comb plate rows (reflecting divergent evolution of details of mosaic development from the pluripotent stem cnidarian ancestor), a major subgroup being armoured; the two long tentacles with colloblasts, to give a greater reach, evolved substantially later in the palaeozoic after bigger prey evolved (secondarily lost in *Beroe* [1]). The benthic coeloplanids are phylogenetically derived [1] with no role in ctenophore or bilaterian origins.

Rapid divergence of Anthozoa (benthic nematocystous adults) and Ctenophora (pelagic direct developing ciliary feeders; the first) neatly partitioned the Early Cambrian adaptive zone for predating larger prey. By not settling, ctenophores could evolve anal pores at the statocyst pole, enabling more efficient unidirectional ingestion and defecation currents independently of unianal bilateria, allowing secondary biradial gut symmetry by losing the siphonoglyph (convergently with scleractinian corals). Like ctenophores, early adult anthozoa probably relied on ciliary feeding (often helped by mucus secretion as in scleractinia). More complex barbed nematocysts (from the likely ancestral atrichous isorhizas) and toxins evolved divergently only after bilateria arose and became cnidarian prey.

## 9. Origin of bilateria and the coelom

The first bilaterian and coelom could have evolved most simply through repercussions of a single key mutation modifying early pharyngeal development of a stem anthozoan polyp. The anthozoan pharynx (stomodaeum) develops separately from the coelenteron cavity by an apical inwardly projecting tissue mass that secondarily develops an inner cavity (e.g. *Renilla*) or by apical invagination (*Alcyonium*); the stomodaeal cavity/invagination joins the coelenteron secondarily when the two separating epithelia at the stomodaeal base degenerate, making a novel opening [82]. A mutation, causing an extended pharynx primordium like that of *Renilla* to fuse basally with the side of the developing coelenteron wall before the breakthrough, would immediately connect the pharyngeal cavity not with the coelenteron but through the body wall to the outside (figure 3). This one-step anal breakthrough [83] would convert the muscular pharynx into a through gut, and transform the coelenteron into a closed coelom, creating a viable ‘hopeful monster’. If the stem anthozoan had one siphonoglyph like octocorals and cerianthid and some actiniarian Zoantharia (often misleadingly called hexacorals), it was already bilaterally symmetric (most likely); if it were biradial with two siphonoglyphs such as some Zoantharia (Antipatharia, some sea anemones), the lateral breakthrough directly made it bilateral. Being hermaphrodite with vegetative reproduction, it could have multiplied enough to invalidate the classic objection against hopeful monsters that they could never find a similar mate.

If the anthozoan that did this was a facultative burrower, as some are, then the coelom could have increased the mechanical efficiency of burrowing (often postulated as its original function) almost without further modification, and separated its mechanical functions from those of a gut. The new through gut could retain digestive and absorptive functions, likely improved by modifying their positional control to regionally differentiate the former pharynx and be suppressed in the former coelenteron, now coelom. By focusing on burrowing and processing ingested sediment, nematocysts were lost and tentacles modified in function to simple mouthparts (or lost in some lineages). Such a radical change would necessarily dramatically affect embryology; unsurprisingly, thereupon two different ways immediately arose to stabilize mouth/anus formation in this protobilaterian: the proterostome/deuterostome bifurcation.

Sequence phylogeny makes it virtually certain that the deuterostome ancestor was non-cephalized, whether a burrower like acorn worm or tentaculate like pterobranchs, possibly colonial like tunicate and salp. All these could readily have arisen from this tentaculate/burrowing intermediate. Lophotrochozoa also appear primitively to have had non-cephalized tentaculate or burrowing forms. The common ancestor of both groups can be argued to have been a tentaculate form, retaining pharyngeal ciliary currents that Anthozoa use in feeding, but a better burrower than burrowing sea anemones. This protobilaterian would be preadapted as ancestor of all major deuterostome and lophotrochozoan groups; acquiring ecdysis and very different mouthparts was more radical, yielding a priapulid-like ecdysozoan ancestor. Site-heterogeneous multigene analyses show that deuterostome acoels lost gut [84,85] and proterostome entoprocts, and independently cephalized Platyhelminthes and Gnathifera (miniaturized interstitial specialists) all lost coeloms independently [84,85]. Whether Xenacoelomorpha are sisters to deuterostomes [84] (thus also lost coeloms) or Nephrozoa [85,86] (so possibly ancestrally acoelomate), they probably arose by simplifying an anthozoan-like ancestor. Xenacoelomorph early divergence, even if true, would not contradict that or justify Hyman's influential antipathy to all ‘coelom early’ theories for bilateria [87].

Although lacking synapses, sponge tissues and embryology are as complex as in Cnidaria [22,88–91]; Hyman [92] wrongly but influentially denied that by labelling sponges a cellular and coelenterates a tissue constructional grade; sponge pattern formation and morphogenesis involve many of the same genes as in other animals, e.g. notch [38]. When I first propounded the ideas summarized in §§2–9 at a 1984 symposium on lower invertebrate origins and relationships [93], only two other speakers took seriously my argument that coeloms evolved in the ancestral bilaterian: Rieger, who had evidence for coelom losses in annelids, and Nielsen who shared my heterodox but right [85] view of Bryozoa as a clade (entoprocts secondarily acoelomate). Almost none thought choanoflagellates relevant to animal origins. Sponge expert Bergquist, the only other participant considering sponges relevant to eumetazoa, agreed that Hyman's dogma that sponges lack proper tissues and are radically simpler than Cnidaria is wrong. The audience of morphologists burst out laughing when I said sequencing mitochondrial genomes of all animal phyla could test my ideas. The symposium volume excluded my invited chapter as a referee called it ‘a farrago of nonsense’, so I took a sabbatical to learn to clone and sequence genes, starting with cnidaria, sponges and choanoflagellates [7]. Cnidarian mitochondrial genomics initiated by my 1987 cloning *Sarcophyton* mitochondrial genome [94] confirmed my 1984 theses that Anthozoa were ancestral to Medusozoa, triploblastic jellyfish ancestral to Hydrozoa, and Eumetozoa ancestrally bilaterally symmetrical and triploblastic [95]. Radial symmetry of Medusozoa and hydrozoan diploblasty is indeed derived. The only primitively radiate animals are sponges.

Hyman's assertion [87] that non-cephalized, often tentaculate bilaterian phyla and classes were obviously all decephalized and simplified by losing mouthparts, sense organs and brains never convinced me. A few, notably barnacles, probably are, but most are not. Arthropod, gnathiferan, mollusc, annelid and vertebrate heads are not morphologically homologous, arguing for independent origins. Their common ancestors more likely than not were non-cephalized tentaculate filter feeders. That all animals have homologous ‘head–tail’ patterning involving Wnt and homeobox gene switches does not make heads homologous. Such genes are just transcriptional switches that connect patterning gradients and downstream cell differentiation and morphogenetic cellular processes that actually make non-homologous structures such as mandibles, chelicerae, rotifer jaws or mollusc radula. Thinking human and grasshopper heads structurally homologous is as bad as calling a vacuum cleaner and light bulb homologous, because identical switches can turn both on. The notion that all animal eyes are homologous, because Pax transcription factors induce all, similarly erroneously confuses organizational levels. Rhodopsin is homologous between proteobacteria and animals, but vertebrate eyes are not structurally homologous with octopus or *Drosophila* eyes; these eyes evolved independently by modifying eukaryote cells (not strictly homologous with the bacteria that invented rhodopsin) and arranging them into contrasting supracellular structures. It is too often overlooked that structural homologies like those of tetrapod limb bones are at a higher level of organization than are transcription factors or building blocks such as collagen that they may share with morphologically non-homologous arthropod or annelid limbs and can often be recognized unambiguously entirely independently of gene sequences; there is almost certainly no ‘pentadactyl-limb gene’. Non-homologous structures (e.g. cilia and muscle; or nematocysts and leg bones) are often built partly of homologous components.

## 10. Vendozoa: diversified presponges?

Rejecting the then prevalent idea that Ediacaran macrofossils antedating the Cambrian explosion included bilateria [96], I argued in 1984 and subsequently [83] that the Cambrian explosion was simply the origin of bilateria, and Vendobionta were all Cnidaria. Critical reevaluation of frondose rangeomorph Vendozoa makes it unlikely they are Cnidaria [97,98]. I now agree with Seilacher [99] that typical modular quilted foliate Vendozoa are not from any extant phyla, though non-foliate approximately 560 Ma old *Haootia* might be a muscular cnidarian impression [81]. However, I reject his idea that Vendozoa are complex, possibly syncytial protists (vendobionts) unrelated to animals [100], his analogy with quilted caps of giant unicellular green alga *Acetabularia* being superficial. *Acetabularia* is not syncytial; its form requiring cell walls is adapted for photosynthesis. Habitat proves that Vendozoa were not generally phototrophs [101]. Syncytial algae such as *Codium* are never quilted. Absorptive feeding by filamentous syncytial fungi like zygomycetes would cease if they evolved that body form. Large fungal fruiting bodies are non-trophic for spore dispersal. Syncytial sponges evolved secondarily from cellular ancestors. The largest protozoan syncytia (myxogastrid Mycetozoa) are naked phagotrophs with no architectural potential to evolve a vendozoan body form, unassignable to any protist group. Vendozoan complexity required extensive connective tissue to make quilt seams as struts supporting two outward facing trophic epithelia. Broken frondule internal structure [102] suggests cellular tissue not syncytia.

That Vendozoa were osmoheterotrophs [103] is implausible; such organisms should be finely divided like a fungal mycelium. Feeding by harbouring chemotrophic bacteria is theoretically possible [104], as in Pogonophora or anaerobic bivalves, but these clearly betray an annelid and mollusc ancestry unlike Vendozoa; both evolved in Lophotrochozoa with a long history of oral/gut feeding (some carnivorous sponges similarly supplement their diet). I do not see how such a symbiosis could have originated and propelled the origin of a complex macrorganismal tissue. Instead, I suggest that quilted Vendozoa were a major presponge radiation (‘Avalon explosion’ [105]) approximately 30 Ma before the AS originated. Rangeomorphs with attachment discs could be bifacial fronds bearing choanocytes on both sides. Dickinsoniids without discs might be flattened presponges living on soft surfaces and differentiated into an upper filter-feeding choanoderm and lower surface without choanocytes (possibly also phagocytosing bacteria beneath it [106]). Often confused with bilateria, dickinsoniid self-mobility is a palaeontological misinterpretation, making it improbable that they are Placozoa [106]; quilt terminal addition does not prove that they are bilateria [107].

It is theoretically possible that Vendozoa arose independently of sponges by evolving a connective tissue in another colonial flagellate group—*Phalansterium* and spongomonads are possibilities that in principle might retain their feeding mechanism after evolving a multicellular differentiated tissue. But I strongly doubt any did, as evolving a multicellular phagotroph with tissues is difficult (see above) except via a flagellate/sponge pathway, and vendozoan timing just before sponges and eumetazoa can hardly be mere coincidence. Vendozoa flourished 580–541 Ma, becoming extinct at the Cambrian explosion approximately 541 Ma. Their reduced disparity and diversity 5 Ma before the Cambrian explosion [108] I attribute to competition from stem sponges with AS, making bilaterian grazing just the final straw that extinguished Vendozoa.

Several simpler, seemingly non-quilted, sessile Ediacaran fossils could also be presponges, e.g. the tubular *Funisia* [109]. The 1 mm *Eocyathispongia* [110] is more reasonably interpreted as a 600 Ma old presponge than as a sponge, as it lacks evidence for an AS, the tiny putative intercellular spaces being insufficient evidence for ostia and channels penetrating the body wall. This interpretation of Ediacaran fossils implies that presponges preceded sponges by scores of millions of years. Oldest undoubted sponges are 535 Ma old hexact spicules, claims for earlier sterols being demosponge-specific being erroneous [111]. Crown sponges must be older, at least as old as Eumetazoa (minimally 541 Ma), but not necessarily older if Eumetazoa evolved from stem sponges. Arguably, spicules evolved independently in calcareous and siliceous sponges by evolving specialized amoeboid sclerocytes only after spicular protection against early pre-molluscan grazers became advantageous; sponge carbonic anhydrases related to those of eumetazoa diversified immensely in Calcarea, aiding calcification [112]. Unique sponge anti-predator secondary metabolites would also have diversified thenceforth. A 40 Ma lag between presponge and sponge origins is reasonable, as rearrangements making an AS were radical, probably mutationally and mechanistically more difficult than the choanoflagellate–presponge transition.

I regard Vendozoa as the oldest phylum of kingdom Animalia, distinct from Porifera, Placozoa and Eumetazoa. I divide it into subphylum Petalonamae [113] for petaloid quilted taxa (even *Kimberella* may belong here [101]) and for non-quilted ones (e.g. *Eocyathispongia*) new subhylum Varisarca: Diagnosis: extinct macroscopic sessile multicells inferred to be ciliary filter feeding phagotrophs with epithelial/ECM organization; body form: variable arrangements of thin sheets, neither arranged in a quilted array (unlike Petalonamae), nor having ostia and internal water channels (unlike Porifera); non-mobile as adults. Etymology *Vari* variable *sarco* Gk flesh signifies variable body forms of epithelioid/ECM presponge fleshy organization.

Vendozoa likely had Wnt/catenin axial patterning and ciliated planktonic larvae for dispersal, as without an AS they could not have easily brooded larvae as most sponges do (perhaps secondarily as protection after coelenterates evolved). If Placozoa are sisters of Eumetazoa as most multigene trees suggest, Placozoa were secondarily simplified by AS or coelenteron loss, evolving neotenously by prolonging the usual larval presettling benthic creeping phase by evolving extracellular digestion of benthic microbes and losing metamorphosis. Only if nested within Eumetazoa (Coelenterata plus bilateria) as some unconvincing trees suggest, need they have lost neurons also, like Myxozoa. Only if branching deeper than sponges and Eumetozoa, which multigene trees mostly exclude, could *Trichoplax* be direct descendants of presponges.

## 11. Cambrian body plan quantum evolution made major new adaptive zones

The Cambrian explosion is the most striking animal example of ultrarapid origins of novel body forms: Simpson's quantum evolution, convincingly attributed to the invasion of previously unexploited major adaptive zones [114]. Some lesser examples (e.g. land invasion generating tetrapods) may have been initiated by behavioural changes allowing entry into pre-existing vacant habitats and associated body plan modifications. Behaviour changed markedly during animal phylum origins—contrast crawling molluscs, burrowing annelids, walking/swimming arthropods and sedentary filtering Bryozoa—but in most cases, mutations creating truly novel body plans effectively simultaneously created body plans and their adaptive zones. Origins of sponges, cnidaria, ctenophores and coelomate bilateria made organisms with novel body plans and thereby new adaptive zones; they cannot sensibly be regarded as responses to environmental change or entry into pre-existing adaptive zones. They were internal non-responsive innovations that worked. Darwin recognized that evolution would necessarily be exceptionally fast immediately a really new organismal type arose. But overawed by Lyellian uniformitarianism, and without understanding how quickly key mutations early in development can suddenly radically change animal phenotypes (exemplified by the above-discussed origins of Porifera, Cnidaria, ctenophores and bilateria), he greatly underestimated how fast it could be, mistakenly supposing animal phyla must have taken eons to evolve from a protozoan and that absence of Precambrian animal fossils meant that the palaeontological record is immensely more incomplete than study of microscopic fossils now shows.

There truly was an Early Cambrian explosion of animal (and protist) phyla, now ecologically and evolutionarily quite easy to understand. Such an explosion is expected for the very reasons that Darwin and Simpson convincingly explained. When a bilaterian with through gut and coelom arose, it created a new competitor-free adaptive zone and was bound to diversify rapidly into all body plans developmentally readily made by simple modifications and able to survive ecologically [83]. It would be a much greater puzzle if all bilaterian phyla had not evolved within 20–30 Ma. It is no longer a mystery why they did: self-creation of radical novelty dramatically alters selective forces and makes novel ancestors with unprecedented evolutionary potential. Animal developmental complexity allows the magnitude of mutational and phenotypic change to be disassociated: small key mutations can effect huge changes. Surprisingly easily, in the right organismal, phylogenetic, developmental and ecological context, they can make new phyla, probably on a similar timescale to the origin and evolutionary radiation of Darwin's finches (2–3 Ma [115]). As stressed above, origins of Cnidaria, ctenophores and bilateria were probably mechanistically much easier than of presponges or sponges, given the intermediates proposed here, so it is now entirely unsurprising that sponges, cnidaria, ctenophores and bilateria appear palaeontologically to have originated in a single geological blink (that makes early sequence tree resolution so hard). That is a nice congruence of palaeontological evidence, sequence tree proportions, and the present organismal evolutionary analysis and synthesis. Once the fundamental triploblastic zoophyte life cycle (pelagic ciliated larva, axial patterning, metamorphosis, triploblastic sessile adults) yielded the first sponge, as soon as the osculum became a mouth its immediate descendants could rapidly generate all other extant animal phyla (body plans and adaptive zones) in a radiative explosion that simultaneously eliminated Vendozoa.

A widespread explanatorily empty speculation that many groups originated long before their objective fossil dates is fuelled by deep uniformitarian prejudices about evolutionary rates that palaeontology long ago refuted, and three other prejudices/biases that synergistically led to the notion of a ‘slow burning fuse’—a journalistic slogan, not critical evolutionary thought, evaluation or synthesis. First is excessive confidence in the certainly false idea of a ‘molecular clock’ and in the reliability of current implementations of oxymoronic ‘relaxed clock’ computer programs [116]. Second is uncritical acceptance of the dubious identity of some fossils used for calibration, driven by palaeontologists’ ‘my fossil is older than yours’ competition [117]. Third is a dearth of coherent imaginative but critical synthesis as done by Darwin and Simpson, often harmfully dismissed as speculation and deterred by journal publishing and refereeing practices, but attempted here instead of merely listing genes from protist genomes potentially significant for originating animal multicellularity.

## 12. Conclusion: from zoophytes to mobile animals

The best way to understand megaevolutionary events is by a coherent synthesis unifying data of every kind using explicit reasoning and well-tested explanatory principles. Haeckel's idea that animals evolved from a protozoan ancestor directly via a gastraea with triploblastic body, mouth, gut and anus, and that the animal archetype was a flatworm-like bilateral mobile predator like us minus coelom and anus must be wrong. A gastraea is far too complicated to evolve in one step. Instead, a choanoflagellate became a triploblastic sponge (arguably in two separate stages), a sponge became an anthozoan cnidarian, stem anthozoa generated pelagic ctenophores and independently an ancestral sessile bryozoan-like bilaterian, whose headless zoophyte descendants independently evolved morphologically contrasting heads through inventing burrowing, crawling or swimming, in annelids, molluscs, arthropods and vertebrates; all acoelomate bilateria arose secondarily by coelom occlusion. Nematocyst-triggered origin of neurons and zoophyte origin of bilateria adumbrated here put sessile headless animals central to eumetazoan and bilateria origins, just as they are to the already widely accepted choanoflagellate-sponge transition (here explicitly elucidated and divided into two possibly temporally distinct phases). All three problems are more deeply illuminated by a unifying zoophyte perspective than by Haeckel's anthropomorphic, self-mobile adult bias. Sessile presponge headless zoophytes with dispersive ciliated larvae were the first animals; muscle-driven mobility is secondary. Heads followed rather than led basic animal innovations. Can a simpler path fit the facts?

## Endnote

1 For Lamarck and Cuvier, zoophyta were the most primitive animal group comprising protozoa, sponges and cnidaria. ‘Zoophyte’ here signifies primitively non-cephalized sessile animal ancestors with ciliated larvae to emphasize (i) that animals probably began and most phyla originated via that organisational grade, not a swimming gastraea-like adult and (ii) that plant-mimicking photosynthetic consortia involving endosymbiotic algae (pro- eu- and meta- [52]) figure largely in the ecology and possibly origins of sponges and anthozoa. Plants and animals evolved independently from zooflagellate ancestors; there never was a transition between them; carnivorous plants that so engaged Erasmus and Charles Darwin as transitional hints [53] are phylogenetically irrelevant red herrings.
